# Differences in Psychiatric Comorbidities and Gender Distribution among Three Clusters of Personality Disorders: A Nationwide Population-Based Study

**DOI:** 10.3390/jcm10153294

**Published:** 2021-07-26

**Authors:** Chih-Wei Hsu, Liang-Jen Wang, Pao-Yen Lin, Chi-Fa Hung, Yao-Hsu Yang, Yu-Ming Chen, Hung-Yu Kao

**Affiliations:** 1 Department of Psychiatry, Kaohsiung Chang Gung Memorial Hospital and Chang Gung University College of Medicine, Kaohsiung 83301, Taiwan; harwicacademia@gmail.com (C.-W.H.); paoyenlin@gmail.com (P.-Y.L.); chifa.hung@gmail.com (C.-F.H.); 2 Department of Computer Science and Information Engineering, National Cheng Kung University, Tainan 70101, Taiwan; 3 Department of Child and Adolescent Psychiatry, Kaohsiung Chang Gung Memorial Hospital and Chang Gung University College of Medicine, Kaohsiung 83301, Taiwan; wangliangjen@gmail.com; 4 Institute for Translational Research in Biomedical Sciences, Kaohsiung Chang Gung Memorial Hospital, Kaohsiung 83301, Taiwan; 5 College of Humanities and Social Sciences, National Pingtung University of Science and Technology, Pingtung 91201, Taiwan; 6 Health Information and Epidemiology Laboratory, Chang Gung Memorial Hospital, Chiayi County 613016, Taiwan; r95841012@ntu.edu.tw; 7 Department of Traditional Chinese Medicine, Chang Gung Memorial Hospital, Chiayi County 613016, Taiwan; 8 School of Traditional Chinese Medicine, College of Medicine, Chang Gung University, Taoyuan 333323, Taiwan

**Keywords:** comorbidities, gender difference, personality disorders, population-based

## Abstract

Personality disorders (PDs) are grouped into clusters A, B, and C. However, whether the three clusters of PDs have differences in comorbid mental disorders or gender distribution is still lacking sufficient evidence. We aim to investigate the distribution pattern across the three clusters of PDs with a population-based cohort study. This study used the Taiwan national database between 1995 and 2013 to examine the data of patients with cluster A PDs, cluster B PDs, or cluster C PDs. We compared the differences of psychiatric comorbidities classified in the *Diagnostic and Statistical Manual of Mental Disorders, fifth edition* across the three clusters of PDs. Moreover, we formed gender subgroups of the three PDs to observe the discrepancy between male and female. Among the 9845 patients, those with cluster A PDs had the highest proportion of neurodevelopmental disorders, schizophrenia and neurocognitive disorders, those with cluster B PDs demonstrated the largest percentage of bipolar disorders, trauma and stressor disorders, feeding and eating disorders, and substance and addictive disorders, and those with cluster C PDs had the greatest proportion of depressive disorders, anxiety disorders, obsessive–compulsive disorders, somatic symptom disorders, and sleep–wake disorders. The gender subgroups revealed significant male predominance in neurodevelopmental disorders and female predominance in sleep–wake disorders across all three clusters of PDs. Our findings support that some psychiatric comorbidities are more prevalent in specified cluster PDs and that gender differences exist across the three clusters of PDs. These results are an important reference for clinicians who are developing services that target real-world patients with PDs.

## 1. Introduction

Personality disorders (PDs) are a class of mental disorders that represent maladaptive self (identity and self-direction) and interpersonal (empathy and intimacy) functioning [1]. They are usually associated with high costs to society and negatively influence progress when treating other mental disorders [2]. PDs are classified into three clusters with different characteristics: cluster A PDs (paranoid, schizoid, and schizotypal) representing odd or eccentric thinking [3], cluster B PDs (antisocial, borderline, histrionic, and narcissistic) manifesting dramatic, overly emotional, or unpredictable behavior [4], and cluster C PDs (avoidant, dependent, and obsessive–compulsive) behaving anxiously and fearfully [5].

PDs have a high prevalence of other mental disorders, ranging from 4.5 to 100 percent, and clinicians may find it difficult to access or provide appropriate management for patients with personality disorder and comorbid mental disorders [6]. It may be because individuals with specified PDs comorbid with certain mental disorders are found to have greater severity and dysfunction [7], which affects treatment outcome. For example, patients with cluster A or cluster B PDs had significantly fewer treatment gains on anxiety disorders than cluster C PDs [8]. Therefore, determining the frequency of psychiatric comorbidities in different PDs may provide clinical insights for further potential treatments. Previous studies have already reported the proportion of comorbidities across the three clusters of PDs, including anxiety disorders [9] and mood disorders [10]. However, they have not yet investigated other mental disorders, such as neurodevelopmental disorders and sleep–wake disorders.

One issue related to the three clusters of PDs has not yet been completely explored. Previous study has shown that cluster A PDs (schizoid, schizotypal, and paranoid) are more often diagnosed in men, and cluster B PDs (borderline and histrionic) may occur more often in women [11]. If the three clusters of PDs are comorbid with other mental disorders, whether they show the same gender differences is still unknown. For example, neurodevelopmental disorders usually demonstrate male predominance [12], but whether the gender pattern is still observed when individuals are classified into different cluster PDs remains unknown.

Relevant literature is still scarce, especially evidence from large national medical databases. Previous research has shown that real-world evidence is very relevant from an external validity perspective, and it recruits a wider patient population to provide results for clinical practice [13]. To fill the research gap, we aimed to use the Taiwan nationwide health insurance database between 1995 and 2013 to investigate the psychiatric comorbidities across the three clusters of PDs. We also analyzed the gender difference among those PDs.

## 2. Methods

### 2.1. Data Source

We used the Taiwan National Health Insurance Database (NHIRD) to obtain data. The NHIRD is derived from the reimbursement medical claims records from Taiwan’s NHI program, which was implemented in 1995 as the sole payer for healthcare services and covers 99% of Taiwan’s population. We linked the NHIRD and extracted the data of the cohort of individuals born between 1 January 1900 and 31 December 2013, which included the insured’s personal information, including gender and clinical diagnostic codes (*International Classification of Diseases, Ninth Revision, Clinical Modification*; ICD-9-CM). All information from the NHIRD that could potentially be used to identify individual patients was anonymized to ensure confidentiality.

### 2.2. Study Population

We included patients with a diagnosis of cluster A PDs (ICD: 301.0, 301.20, 301.22), cluster B PDs (ICD: 301.50, 301.7, 301.81, 301.83), or cluster C PDs (ICD: 301.4, 301.6, 301.82) during the period between 1 January 1995 and 31 December 2013. To ensure diagnostic validity, patients were defined as an ICD code of the same cluster PDs based on the diagnostic interviews and clinical judgments of psychiatrists in inpatient or outpatient settings at least twice; such a definition was adopted in a previous study [14]. For each patient, the date of diagnosis (age at diagnosis) was defined as the first day of the diagnosis of PDs. Furthermore, we excluded patients with an unknown gender status, diagnoses of other specified PDs or unspecified PDs, and diagnoses of more than one cluster PDs. For example, a patient with diagnoses of cluster A and cluster C at a different time within the study period would have been excluded.

### 2.3. Psychiatric Comorbidities

Psychiatric comorbidities were defined as all psychiatric diagnoses between 1 January 1995 and the date of the last diagnosis of PDs (the study follow-up duration). We selected categories of the *Diagnostic and Statistical Manual of Mental Disorders, fifth edition* (DSM-5) [15] as the diagnostic outcomes of psychiatric comorbidities because the items considered for the main classification in the DSM-5 are more detailed than those in the ICD. For example, bipolar and depressive disorders, which are separate entities in the DSM-5, are both classified as mood disorders in the ICD [15]. We excluded substance- or medication-induced mental disorders, as well as mental disorders due to another medical condition. Furthermore, we excluded patients with an unknown gender status, diagnoses of other specified PDs or unspecified PDs, and diagnoses of more than one cluster PDs. The major psychiatric comorbidities (18 mental disorders) with diagnostic codes are shown as follows and in Supplementary Table S1: (1) neurodevelopmental disorders; (2) schizophrenia; (3) bipolar disorders; (4) depressive disorders; (5) anxiety disorders; (6) obsessive–compulsive disorders; (7) trauma and stressor disorders; (8) dissociative disorders; (9) somatic symptom disorders; (10) feeding and eating disorders; (11) elimination disorders; (12) sleep–wake disorders; (13) sexual dysfunctions; (14) gender dysphoria; (15) disruptive, impulse-control, and conduct disorders (DICDs); (16) substance and addictive disorders; (17) neurocognitive disorders; and (18) paraphilic disorders. For diagnostic validity in this study, the diagnosis of such psychiatric comorbidity was defined as at least two diagnoses of the same psychiatric disorder by psychiatrists.

### 2.4. Statistical Methods and Sensitivity Analyses

To examine the differences in gender, age at diagnosis, and psychiatric comorbidities between cluster A, cluster B, and cluster C PDs, we used the one-way analysis of variance (ANOVA) [16]. Furthermore, we performed subgroup analysis (male and female groups) and adopted Pearson’s chi-squared test (for category variables) or independent sample t-test (for continuous variables) to evaluate gender differences in age and comorbidities among the same cluster PDs. Gender subgroup analysis was then carried out to examine the differences within the same cluster PDs. In addition, we also performed two sensitivity analyses to evaluate the robustness of our results. First, to improve diagnostic stability and validity, we increased the thresholds for the inclusion criteria of diagnosis by psychiatrists to at least four times, not twice as in the primary definition [17]. Then, we repeated the above primary analysis. Second, to evaluate factor structure of the three clusters of PDs, we performed a principal component analysis (PCA) for each psychiatric comorbidity of the PDs. Components that yielded eigenvalues exceeding 1.00 were considered important factors. Moreover, we also drew an Upset plot for overlapping psychotic comorbidities in the same cluster PDs [18]. All analyses were conducted with SAS 9.4 software (SAS Institute Inc., Cary, NC, USA). In this study, we used multiple tests for the various cluster PDs, and the results were considered statistically significant if the two-tailed *p*-value was less than 0.001 (approximating a Bonferroni correction; the exact values are shown in the footnotes of Table and Supplementary Table). We also reported the F value of ANOVA, the *t* value of *t*-test, and the X^2^ of chi-squared test.

## 3. Results

**Figure 1:**
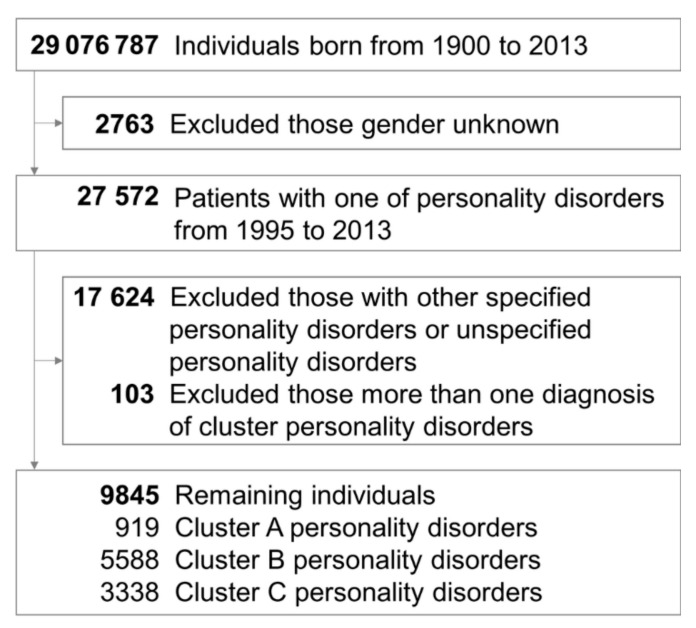
Flowchart showing the selection procedure of study subjects.

Figure 1 shows the processes used to select the patients in the Taiwanese study cohort. We ultimately identified 9845 cases with a specified cluster of PDs between 1995 and 2013. These cases were followed until the last psychiatric outpatient or inpatient service, with a mean follow-up time of 12.55 years (standard deviation: 4.14). The basic characteristics of the included individuals are described in Table 1. Of those patients, 919 were in the cluster A group, 5588 were in the cluster B group, and 3338 were in the cluster C group. In the three groups, males were predominant in cluster A PDs (62.35%), whereas females were predominant in cluster B PDs (69.11%) and C PDs (55.54%). As for psychiatric comorbidities, neurodevelopmental disorders (11.97%), schizophrenia (39.06%), and neurocognitive disorders (11.32%) were the most common comorbidities in cluster A PDs; bipolar disorders (22.10%), trauma and stressor disorders (21.76%), feeding and eating disorders (5.82%), and substance and addictive disorders (22.15%) were most frequently accompanied with cluster B PDs; and depressive disorders (65.43%), anxiety disorders (74.81%), obsessive–compulsive disorders (17.38%), somatic symptom disorders (9.14%), and sleep–wake disorders (72.23%) mostly cooccurred with cluster C PDs. Furthermore, depressive disorders (47.12% to 65.43%), anxiety disorders (51.18% to 74.81%), and sleep–wake disorders (52.88% to 72.23%) were the three most common psychiatric comorbidities of all three clusters of PDs. Meanwhile, the psychiatric comorbidities of dissociative disorders, elimination disorders, sexual dysfunctions, gender dysphoria, DICDs, and paraphilic disorder were no more than 5%. Moreover, Supplementary Table S2 shows the sensitivity analysis after adjusting the diagnostic threshold of PDs (4 times). The distribution of gender (male vs. female) and psychiatric comorbidities (most comorbid with what kind of PDs) were all the same as the primary outcomes. Supplementary Tables S3–S5 and Supplementary Figures S1–S3 show the PCA of three clusters of PDs, and there were more than eight factors with eigenvalues greater than 1.00. Moreover, Supplementary Figures S4–S6 present the Upset plots of three clusters of PDs. Depressive disorders, anxiety disorders, and sleep–wake disorders were the most overlapping psychiatric comorbidities.

Table 2 lists the gender difference in three clusters of PDs. For psychiatric comorbidities, we found male predominance in childhood-onset disorders (neurodevelopmental disorders and DICDs), schizophrenia, trauma and stressor disorders, substance and addictive disorders, obsessive–compulsive disorders, and sexual disorders (sexual dysfunctions, gender dysphoria, and paraphilic disorders) across all three clusters of PDs, with significance observed in neurodevelopmental disorders. Additionally, we found female predominance in neurocognitive disorders, mood disorders (bipolar disorders and depressive disorders), anxiety disorders, eating and eliminating disorders (feeding and eating disorders and elimination disorders), sleep–wake disorders, and dissociative disorders. Of those, sleep–wake disorders reached the level of a significant difference in all three clusters of PDs. In Supplementary Table S6, we found that with the exception of schizophrenia, neurocognitive disorders, and depressive disorders, all the other disorders revealed the same consistent male or female predominance across the three clusters of PDs.

## 4. Discussion

We used the Taiwanese national health insurance database to perform a comprehensive assessment of both the overall difference and gender difference in psychiatric comorbidities among the three clusters of PDs. Among the three PDs, we found cluster A PDs to have the highest proportion of neurodevelopmental disorders, schizophrenia, and neurocognitive disorders, cluster B PDs to have the largest percentage of bipolar disorders, trauma and stressor disorders, feeding and eating disorders, and substance and addictive disorders, and cluster C PDs to have the maximum proportion of depressive disorders, anxiety disorders, obsessive–compulsive disorders, somatic symptom disorders, and sleep–wake disorders. Considering the gender subgroup of the three PDs, we observed significant male predominance in neurodevelopmental disorders and female predominance in sleep–wake disorders across all three clusters of PDs.

Although NHIRD is one of the core data resources of the Asian Pharmacoepidemiology Network and has a high utilization rate in conducting international comparative studies [19], the prevalence of PD in our study was lower than in the previous meta-analysis (our study: 0.1%; meta-analysis: 12.2%) [20]. Different study designs can explain the difference in prevalence. The meta-analysis is based on data from community samples [20]; Taiwan’s NHIRD is a reimbursement of medical expenses, which means that subjects with PD diagnosis in the database are indeed patients who need medical treatment due to their serious disease course. Therefore, the subjects with a mild degree of PD may stay in the community mostly and are not included in this database. This may lead to an underestimation of the prevalence of PD diagnosis in our study.

Previous studies have compared the proportion of several psychiatric comorbidities across three clusters of PDs [9,10], and certain findings were in line with those in our study. For example, cluster B PDs mostly co-occurred with bipolar disorders [10], and cluster C PDs frequently dominated with depressive disorders, anxiety disorders, and obsessive–compulsive disorders [9,10]. Furthermore, among the three PDs, we found that individuals with cluster B PDs had a significantly higher proportion of trauma and stressor disorders, feeding and eating disorders, and substance and addictive disorders; those with cluster C PDs demonstrated a higher comorbidity with somatic symptom disorders and sleep–wake disorders; and those with cluster A PDs highly co-occurred with neurodevelopmental disorders, schizophrenia, and neurocognitive disorders. The aforementioned phenomena may be explained as follows: First, they reflect the diagnostic characteristics of the subgroup of the three PDs, such as affective instability and emotional liability in borderline PD (cluster B) and bipolar disorder [21,22], sharing the same symptoms of alexithymia in patients with borderline PD (cluster B) or feeding and eating disorders [23], and there is an overlap of the core symptoms between obsessive–compulsive PD (cluster C) and obsessive–compulsive disorder [24]. Second, they result from some poor social interaction due to the trait of specified cluster PD, such as the intimate connection between antisocial PD (cluster B) and illegal drug abuse [25] and the status of depression and anxiety because of extreme sensitivity to negative evaluation in individuals with avoidant PD (cluster C). Finally, the same pathology of brain structures or genes have been detected in these disorders. For example, schizotypal PD (cluster A), schizophrenia, and attention deficit hyperactivity disorder (neurodevelopmental disorders) shared social and attentional deficits based on the dysfunction of cortical, temporal, and prefrontal brain areas [26,27] and dopamine-related genes [28]; compared to healthy controls, schizotypal PD (cluster A) had a significant function decline in visual–spatial working memory [29], which may result in the vulnerability of cognitive impairment (neurocognitive disorders).

The trend of gender predominance seemed relatively consistent among the three PDs. For examples, males were highly comorbid with childhood-onset disorder (neurodevelopmental disorders and DICDs) and sexual disorders (sexual dysfunctions, gender dysphoria, and paraphilic disorders) compared to females; on the other hand, females were more likely to have eating and eliminating disorders (feeding and eating disorders and elimination disorders) and sleep–wake disorders. The gender difference also appeared to have the same trend in patients with/without PDs, such as males with a higher frequency of autism and attention deficit hyperactivity disorder [30,31] and females with a higher prevalence of insomnia [32]. Therefore, our study indicates that whether the individuals had cluster A, cluster B, or cluster C PDs, their gender difference of comorbidities persistently coincided with current evidence of mental disorders.

The strengths of our study are that it is a population-based survey with good follow-up throughout and a large sample size. We also included the diagnosis of PDs by a psychiatrist, not based on a self-rated questionnaire, and further reinforced diagnostic validity by elevating the threshold of the diagnostic number. Nevertheless, several limitations should be considered when interpreting our results. First, we categorized the PDs and psychiatric comorbidities according to the main group of DSM-5 and did not perform subgroup analysis, for example, only three clusters of PDs, not paranoid, schizoid, schizotypal, antisocial, borderline, histrionic, narcissistic, avoidant, dependent, or obsessive–compulsive PD, and only neurodevelopmental disorders, not autism, attention deficit hyperactivity disorder, or intellectual disability. As a result, our study was not able to explore the potential differences in these subgroups. Second, this study was subject to the usual limitations of a retrospective analysis of reimbursement data, so many important clinical characteristics, such as patients’ socioeconomic status, family function and history, or the severity of the symptoms, were not available. Therefore, some bias may be present in the explanation of the data. Third, after excluding subjects without a specific cluster of PD, 1.04% of patients with a PD diagnosis had another, different cluster PD diagnosis, and this prevalence was lower than that in the previous study [33]. It may be due to the definition in the study, with at least two diagnoses, which leaves us with the analysis of this study being the most prominent patient in personality pathology. Finally, although psychiatrists confirmed the diagnosis of PDs at least twice, the lack of DSM-5 criteria or validated scoring scales (e.g., Structured Clinical Interview for DSM-5 Disorders—Clinician Version) was also a limitation [33].

## 5. Conclusions

According to nationwide data, some psychiatric comorbidities classified in the DSM-5 are more prevalent in specified cluster PDs, and gender differences may be present across three clusters of PDs. Our study provides an important real-world reference to remind clinicians of the common associations between psychiatric comorbidities and different PDs. In this way, clinicians can diagnose potential comorbidities of PDs during early visits, and then plan individual treatment services as early as possible.

## Figures and Tables

**Table 1:** Characteristics of individuals in cluster A, cluster B, and cluster C personality disorder groups.

Characteristics	Cluster A *N* = 919	Cluster B *N* = 5588	Cluster C *N* = 3338	F	*p*-Value
Gender				212.68	**<0.0001**
Male	573 (62.35)	1726 (30.89)	1484 (44.46)		
Female	346 (37.65)	3862 (69.11)	1854 (55.54)		
Age at diagnosis, years	38.49 ± 20.74	27.71 ± 9.76	43.17 ± 16.00	1437.36	**<0.0001**
Psychiatric comorbidities					
Neurodevelopmental disorders	110 (11.97)	338 (6.05)	134 (4.01)	41.55	**<0.0001**
Schizophrenia	359 (39.06)	575 (10.29)	465 (6.08)	427.83	**<0.0001**
Neurocognitive disorders	104 (11.32)	31 (0.55)	100 (3.00)	208.58	**<0.0001**
Bipolar disorders	96 (10.45)	1235 (22.10)	333 (9.98)	127.59	**<0.0001**
Trauma and stressor disorders	128 (13.93)	1216 (21.76)	545 (16.33)	29.10	**<0.0001**
Feeding and eating disorders	11 (1.20)	325 (5.82)	49 (1.47)	63.29	**<0.0001**
Substance and addictive disorders	57 (6.20)	1238 (22.15)	206 (6.17)	250.73	**<0.0001**
Depressive disorders	433 (47.12)	3260 (58.34)	2184 (65.43)	55.76	**<0.0001**
Anxiety disorders	486 (52.88)	2860 (51.18)	2497 (74.81)	263.84	**<0.0001**
Obsessive–compulsive disorders	119 (12.95)	347 (6.21)	580 (17.38)	144.13	**<0.0001**
Somatic symptom disorders	50 (5.44)	230 (4.12)	305 (9.14)	47.81	**<0.0001**
Sleep–wake disorders	486 (52.88)	3572 (63.92)	2411 (72.23)	69.95	**<0.0001**
Dissociative disorders	9 (0.98)	57 (1.02)	13 (0.39)	5.42	0.0044
Elimination disorders	33 (3.59)	61 (1.09)	108 (3.24)	30.04	**<0.0001**
Sexual dysfunctions	12 (1.31)	29 (0.52)	63 (1.89)	19.09	**<0.0001**
Gender dysphoria	3 (0.33)	16 (0.29)	6 (0.18)	0.57	0.5635
Disruptive, impulse-control, and conduct disorders	10 (1.09)	59 (1.06)	14 (0.42)	5.43	0.0044
Paraphilic disorders	4 (0.44)	8 (0.14)	9 (0.27)	1.96	0.1412

Data are expressed as *N* (%) or the mean ± standard deviation. Bold type indicates statistical significance (*p* < 0.001), the exact value of Bonferroni correction is 0.0024 (0.05/21). Bottom line indicates that the proportion of all control groups is less than 5%, and its statistical power may be weak.

**Table 2:** Gender differences in cluster A, cluster B, and cluster C personality disorder groups.

Characteristics	Cluster A Male *N* = 573	Female *N* = 346	*t* or X^2^	*p*	Cluster B Male *N* = 1726	Female *N* = 3862	*t* or X^2^	*p*	Cluster C Male *N* = 1484	Female *N* = 1854	*t* or X^2^	*p*
Age at diagnosis, years	33.46 ± 18.51	46.82 ± 21.55	9.95	**<0.0001**	25.84 ± 9.88	28.54 ± 9.59	9.63	**<0.0001**	41.93 ± 16.95	44.16 ± 15.13	4.02	**<0.0001**
Psychiatric comorbidities												
Neurodevelopmental disorders	87 (15.18)	23 (6.65)	14.92	0.0001	225 (13.04)	113 (2.93)	214.55	**<0.0001**	91 (6.13)	43 (2.32)	31.10	**<0.0001**
Disruptive, impulse-control, and conduct disorders	8 (1.40)	2 (0.58)	1.34	0.2468	31 (1.80)	28 (0.73)	13.10	0.0003	11 (0.74)	3 (0.16)	6.63	0.0101
Schizophrenia	226 (39.44)	133 (38.44)	0.09	0.7629	195 (11.30)	380 (9.84)	2.75	0.0974	115 (7.75)	88 (4.75)	13.01	0.0003
Trauma and stressor disorders	91 (15.88)	37 (10.69)	4.84	0.0278	495 (28.68)	721 (18.67)	70.20	**<0.0001**	250 (16.85)	295 (15.91)	0.53	0.4678
Substance and addictive disorders	45 (7.85)	12 (3.47)	7.13	0.0076	402 (23.29)	836 (21.65)	1.87	0.1715	118 (7.95)	88 (4.75)	14.62	0.0001
Obsessive–compulsive disorders	88 (15.36)	31 (8.96)	7.83	0.0051	108 (6.26)	239 (6.19)	0.01	0.9216	336 (22.64)	244 (13.16)	51.61	**<0.0001**
Sexual dysfunctions	12 (2.09)	0 (0.00)	7.34	0.0067	23 (1.33)	6 (0.16)	32.02	**<0.0001**	54 (3.64)	9 (0.49)	44.26	**<0.0001**
Gender dysphoria	3 (0.52)	0 (0.00)	1.82	0.1776	11 (0.64)	5 (0.13)	10.78	0.0010	5 (0.34)	1 (0.05)	3.68	0.0551
Paraphilic disorders	3 (0.52)	1 (0.29)	0.27	0.6008	4 (0.23)	4 (0.10)	1.37	0.2417	7 (0.47)	2 (0.11)	4.06	0.0440
Neurocognitive disorders	39 (6.81)	65 (18.79)	30.85	**<0.0001**	8 (0.46)	23 (0.60)	0.38	0.5392	42 (2.83)	58 (3.13)	0.25	0.6155
Bipolar disorders	53 (9.25)	43 (12.43)	2.33	0.1270	258 (14.95)	977 (25.30)	74.22	**<0.0001**	123 (8.29)	210 (11.33)	8.47	0.0036
Depressive disorders	267 (46.60)	166 (47.98)	0.16	0.6847	819 (47.45)	2441 (63.21)	121.83	**<0.0001**	925 (62.33)	1259 (67.91)	11.33	0.0008
Anxiety disorders	282 (49.21)	204 (58.96)	8.22	0.0041	623 (36.10)	2237 (57.92)	227.48	**<0.0001**	1071 (72.17)	1426 (76.91)	9.85	0.0017
Feeding and eating disorders	3 (0.52)	8 (2.31)	5.84	0.0157	12 (0.70)	313 (8.10)	119.55	**<0.0001**	7 (0.47)	42 (2.27)	18.33	**<0.0001**
Elimination disorders	15 (2.62)	18 (5.20)	4.16	0.0413	10 (0.58)	51 (1.32)	6.07	0.0138	37 (2.49)	71 (3.83)	4.70	0.0301
Sleep–wake disorders	258 (45.03)	228 (65.90)	37.71	**<0.0001**	797 (46.18)	2775 (71.85)	341.05	**<0.0001**	976 (65.77)	1435 (77.40)	55.60	**<0.0001**
Dissociative disorders	2 (0.35)	7 (2.02)	6.23	0.0125	9 (0.52)	48 (1.24)	6.15	0.0131	4 (0.27)	9 (0.49)	0.99	0.3197
Somatic symptom disorders ^a^	27 (4.71)	23 (6.65)	1.57	0.2101	52 (3.01)	178 (4.61)	7.70	0.0055	143 (9.64)	162 (8.74)	0.80	0.3708

Data are expressed as *N* (%) or the mean ± standard deviation. Bold type indicates statistical significance (*p* < 0.001), the exact value of Bonferroni correction is 0.0025 (0.05/20). Bottom line indicates that the proportion of all control groups is less than 5%, and its statistical power may be weak. ^a^ The proportion is not consistently predominant for one gender (male or female) across three clusters of personality disorders.

## Data Availability

The data that support the findings of this study are not publicly available but can be accessed with permission from the National Health Insurance Administration, Ministry of Health and Welfare in Taiwan.

## Supplementary Materials

The following are available online at https://www.mdpi.com/article/10.3390/jcm10153294/s1, Figure S1: Scree plot and variance explained of cluster A personality disorder; Figure S2: Scree plot and variance explained of cluster B personality disorder; Figure S3: Scree plot and variance explained of cluster C personality disorder; Figure S4: Upset plot of cluster A personality disorder; Figure S5: Upset plot of cluster B personality disorder; Figure S6: Upset plot of cluster C personality disorder; Table S1: Codes of International Classification of Diseases, Ninth Revision (ICD-9) for each mental disorder in the Diagnostic and Statistical Manual of Mental Disorders, Fifth Edition (DSM-5); Table S2: Characteristics of individuals in cluster A, cluster B, and cluster C personality disorder groups after sensitivity analysis; Table S3: The components produced by principal component analysis of cluster A personality disorder subgroups; Table S4: The components produced by principal component analysis of cluster B personality disorder subgroups; Table S5: The components produced by principal component analysis of cluster C personality disorder subgroups; Table S6: Gender differences in cluster A, cluster B, and cluster C personality disorder groups after sensitivity analysis.
