# Supplementary material for: Differences in Psychiatric Comorbidities and Gender Distribution among Three Clusters of Personality Disorders: A Nationwide Population-Based Study
Source: J Clin Med. 2021 Jul 26;10(15):3294. doi: 10.3390/jcm10153294 (PMC8347782; doi:10.3390/jcm10153294)
Supplement: Supplementary file 1 [file jcm-10-03294-s001.zip › jcm-1274431-supplementary.pdf]

Table S1. Codes of International Classification of Diseases, Ninth Revision (ICD-9) for each mental disorder in the Diagnostic and Statistical Manual of Mental Disorders, Fifth Edition (DSM-5).

Diagnostic categories (DSM-5)	Codes (ICD-9)
Neurodevelopmental disorders	299, 307.2, 307.3, 307.9, 314, 315, 317, 318, 319
Schizophrenia	295, 297.0, 297.1, 297.2, 298.3, 298.4, 298.8, 298.9
Bipolar disorders	296.0, 296.1, 296.4, 296.5, 296.6, 296.7, 296.80, 296.89, 301.13
Depressive disorders[a]	296.2, 296.3, 296.99, 300.4, 311, 625.4
Anxiety disorders	300.00, 300.01, 300.02, 300.09, 300.2, 309.21, 313.23
Obsessive-compulsive disorders	300.3, 300.7, 312.39, 698.4
Trauma and stressor disorders	308.3, 309.0, 309.24, 309.28, 309.3, 309.4, 309.81, 309.89, 309.9, 313.89
Dissociative disorders	300.12, 300.13, 300.14, 300.15, 300.6
Somatic symptom and related disorders	300.7, 300.8, 300.11, 300.19, 316
Feeding and eating disorders	307.1, 307.5
Elimination disorders	307.6, 307.7, 787.60, 788.30, 788.39
Sleep-wake disorders	307.4, 327, 347, 780.5
Sexual dysfunctions	302.7
Gender dysphoria	302.6, 302.85
Disruptive, impulse-control, and conduct disorders	312.32, 312.33, 312.34, 312.8, 312.9, 313.81
Substance and addictive disorders	291.81, 291.4, 292.0, 292.2, 303, 304, 305, 312.31
Neurocognitive disorders	290.1, 290.2, 290.3, 290.4, 294.1, 294.2, 331.0, 331.1, 331.2, 331.8, 331.9
Paraphilic disorders	302.2, 302.3, 302.4, 302.81, 302.82, 302.83, 302.84, 302.89, 302.9

[a] During the study period, if someone had an initial diagnosis of depressive disorder that was subsequently changed to bipolar disorders, the patient was excluded from the category of depressive disorders.

Table S2. Characteristics of individuals in cluster A, cluster B, and cluster C personality disorder groups after sensitivity analysis.

Characteristics	Cluster A N = 570	Cluster B N = 3786	Cluster C N = 2340	F	*p*
Gender				192.14	**<.0001**
Male	366 (64.21)	1079 (28.50)	1057 (45.17)		
Female	204 (35.79)	2707 (71.50)	1283 (54.83)		
Age at diagnosis, years	38.32 ± 20.51	28.28 ± 9.98	43.86 ± 15.63	1013.35	**<.0001**
Psychiatric comorbidities					
Neurodevelopmental disorders	66 (11.58)	195 (5.15)	74 (3.16)	34.70	**<.0001**
Schizophrenia	237 (41.58)	418 (11.04)	142 (6.07)	303.85	**<.0001**
Neurocognitive disorders	70 (12.28)	22 (0.58)	75 (3.21)	149.50	**<.0001**
Bipolar disorders	62 (10.88)	913 (24.12)	254 (10.85)	99.29	**<.0001**
Trauma and stressor disorders	81 (14.21)	824 (21.76)	372 (15.90)	21.02	**<.0001**
Feeding and eating disorders	8 (1.40)	233 (6.15)	35 (1.50)	46.14	**<.0001**
Substance and addictive disorders	37 (6.49)	874 (23.09)	159 (6.79)	172.16	**<.0001**
Depressive disorders	281 (49.30)	2207 (58.29)	1575 (67.31)	42.03	**<.0001**
Anxiety disorders	307 (53.86)	1999 (52.80)	1791 (76.54)	188.60	**<.0001**
Obsessive-compulsive disorders	87 (15.26)	252 (6.66)	425 (18.16)	102.27	**<.0001**
Somatic symptom disorders	34 (5.96)	157 (4.15)	221 (9.44)	35.52	**<.0001**
Sleep-wake disorders	302 (52.98)	2498 (65.98)	1727 (73.80)	51.39	**<.0001**
Dissociative disorders	4 (0.70)	42 (1.11)	8 (0.34)	5.37	.0047
Elimination disorders	23 (4.04)	45 (1.19)	73 (3.12)	18.80	**<.0001**
Sexual dysfunctions	8 (1.40)	22 (0.58)	56 (2.39)	18.86	**<.0001**
Gender dysphoria	3 (0.53)	10 (0.26)	2 (0.09)	2.31	.0997
Disruptive, impulse-control, and conduct disorders	6 (1.05)	42 (1.11)	6 (0.26)	6.82	.0011
Paraphilic disorders	4 (0.70)	5 (0.13)	9 (0.38)	3.90	.0203

Data are expressed as N (%) or the mean ± standard deviation. Bold type indicates statistical significance ($p < 0.001$), the exact value of Bonferroni correction is 0.0024 (0.05/21). Bottom line indicates that the proportion of all control groups is less than 5%, and its statistical power may be weak.

Table S3. The components produced by principal component analysis of cluster A personality disorder subgroups.

Characteristics	Factor 1	Factor 2	Factor 3	Factor 4	Factor 5	Factor 6	Factor 7	Factor 8
Psychiatric comorbidities								
Anxiety disorders	0.709	0.083	0.076	0.040	0.030	0.103	0.119	0.079
Sleep-wake disorders	0.699	-0.060	-0.119	0.192	0.131	0.134	0.044	0.079
Bipolar disorders	0.319	0.660	-0.270	0.089	-0.266	0.041	0.180	0.010
Trauma and stressor disorders	0.126	0.473	-0.092	-0.075	0.305	0.165	-0.046	-0.290
Depressive disorders	0.289	-0.606	0.325	-0.169	0.321	0.185	-0.068	-0.125
Paraphilic disorders	-0.133	0.139	0.563	0.520	-0.125	0.146	-0.047	0.007
Gender dysphoria	-0.098	0.067	0.490	0.414	-0.386	0.188	-0.025	-0.091
Obsessive-compulsive disorders	0.245	0.131	0.423	-0.134	0.050	-0.388	0.249	-0.056
Neurocognitive disorders	-0.084	-0.249	-0.378	0.557	0.179	-0.251	-0.002	-0.013
Elimination disorders	0.057	0.149	-0.161	0.531	0.467	-0.033	-0.209	-0.103
Disruptive, impulse-control, and conduct disorders	-0.249	0.247	0.349	0.025	0.440	0.126	0.060	0.277
Somatic symptom disorders	0.394	0.063	0.308	0.062	0.184	-0.492	0.039	-0.081
Feeding and eating disorders	0.055	-0.086	-0.025	-0.052	0.000	0.428	0.600	-0.290
Dissociative disorders	0.097	-0.128	0.002	0.054	-0.080	-0.194	0.332	0.665
Substance and addictive disorders	0.328	-0.063	0.117	-0.126	-0.026	0.210	-0.359	-0.011
Sexual dysfunctions	0.164	0.066	-0.063	-0.005	0.018	0.403	-0.291	0.501
Schizophrenia	0.208	0.173	0.085	-0.238	-0.262	-0.235	-0.455	-0.067
Neurodevelopmental disorders	-0.320	0.376	0.152	-0.304	0.414	0.017	0.033	0.137

Table S4. The components produced by principal component analysis of cluster B personality disorder subgroups.

Characteristics	Factor 1	Factor 2	Factor 3	Factor 4	Factor 5	Factor 6	Factor 7	Factor 8
Psychiatric comorbidities								
Bipolar disorders	0.770	-0.394	-0.202	-0.089	-0.061	-0.024	0.055	0.069
Depressive disorders	-0.627	0.638	0.139	-0.017	0.069	0.004	0.009	-0.048
Sleep-wake disorders	0.444	0.584	-0.131	0.025	0.142	0.101	-0.018	-0.096
Obsessive-compulsive disorders	0.221	0.076	0.654	-0.209	-0.157	-0.024	0.056	0.116
Sexual dysfunctions	0.014	0.079	0.116	0.426	-0.226	-0.239	0.271	0.386
Feeding and eating disorders	0.193	0.020	0.048	-0.551	0.108	-0.029	0.244	0.099
Dissociative disorders	0.057	-0.018	0.170	-0.058	0.609	-0.447	0.018	-0.039
Gender dysphoria	-0.025	-0.090	0.129	-0.009	0.554	0.067	-0.240	0.291
Paraphilic disorders	0.006	0.010	0.073	-0.037	0.197	0.580	0.656	-0.074
Disruptive, impulse-control, and conduct disorders	0.010	-0.136	0.323	0.018	-0.123	0.003	-0.068	-0.679
Elimination disorders	0.090	0.059	0.051	-0.104	-0.101	0.426	-0.515	0.314
Neurocognitive disorders	0.041	0.056	0.066	0.497	0.057	0.155	0.122	0.217
Trauma and stressor disorders	0.071	-0.269	0.141	0.359	0.270	0.082	-0.185	-0.218
Schizophrenia	0.269	0.048	0.269	0.172	-0.029	-0.311	0.167	0.069
Neurodevelopmental disorders	-0.060	-0.401	0.362	0.195	0.102	0.289	-0.011	-0.014
Substance and addictive disorders	0.352	0.256	-0.207	0.275	-0.069	-0.011	-0.069	-0.236
Somatic symptom disorders	0.261	0.226	0.499	-0.062	-0.229	0.015	-0.160	0.033
Anxiety disorders	0.509	0.487	0.020	0.090	0.216	0.126	-0.053	-0.080

Table S5. The components produced by principal component analysis of cluster C personality disorder subgroups.

Characteristics	Factor 1	Factor 2	Factor 3	Factor 4	Factor 5	Factor 6	Factor 7	Factor 8	Factor 9
Psychiatric comorbidities									
Depressive disorders	0.717	-0.444	-0.067	0.087	0.028	0.123	0.063	0.026	0.001
Bipolar disorders	-0.531	0.656	0.093	0.002	0.075	0.009	-0.020	-0.013	-0.028
Anxiety disorders	0.380	0.546	0.079	0.204	-0.093	-0.020	-0.076	0.046	-0.005
Obsessive-compulsive disorders	0.029	-0.118	0.670	0.145	-0.243	-0.234	0.024	-0.020	-0.243
Schizophrenia	-0.119	-0.038	0.505	0.218	0.138	0.366	0.063	0.256	0.098
Somatic symptom disorders	0.319	0.061	0.490	0.147	-0.127	-0.266	-0.181	-0.112	0.216
Sexual dysfunctions	0.108	0.095	0.123	-0.498	-0.320	0.180	0.252	-0.112	0.046
Dissociative disorders	-0.028	-0.067	0.223	-0.015	0.525	0.258	-0.215	-0.257	0.505
Neurocognitive disorders	0.125	0.022	0.069	-0.374	0.458	-0.211	0.249	0.247	0.178
Paraphilic disorders	-0.018	0.084	0.180	-0.355	-0.334	0.480	0.164	-0.193	0.090
Disruptive, impulse-control, and conduct disorders	-0.057	-0.105	-0.053	0.303	-0.121	0.052	0.619	0.057	0.245
Substance and addictive disorders	0.051	0.200	-0.056	0.439	0.149	0.219	0.458	-0.045	-0.085
Gender dysphoria	-0.072	-0.077	0.072	0.026	0.037	0.339	-0.187	0.677	-0.263
Feeding and eating disorders	-0.041	-0.079	0.087	0.089	0.361	0.055	0.115	-0.495	-0.561
Trauma and stressor disorders	-0.162	0.007	-0.247	0.282	-0.232	-0.183	-0.035	0.036	0.294
Elimination disorders	0.140	0.173	0.139	-0.281	0.204	-0.389	0.348	0.230	-0.109
Neurodevelopmental disorders	-0.412	-0.251	0.174	0.062	-0.029	-0.242	0.148	-0.005	0.193
Sleep-wake disorders	0.518	0.529	-0.076	0.079	0.057	0.050	-0.027	0.001	0.085

Table S6. Gender differences in cluster A, cluster B, and cluster C personality disorder groups after sensitivity analysis.

Characteristics	Cluster A Male N = 366	Female N = 204	t or X^2	*p*	Cluster B Male N = 1079	Female N = 2707	t or X^2	*p*	Cluster C Male N = 1057	Female N = 1283	t or X^2	*p*
Age at diagnosis, years	33.52 ± 17.94	46.94 ± 22.00	7.88	**<0.0001**	26.34 ± 10.21	29.05 ± 9.79	7.60	**<0.0001**	42.93 ± 16.54	44.63 ± 14.80	2.63	**<0.0001**
Psychiatric comorbidities												
Neurodevelopmental disorders	54 (14.75)	12 (5.88)	10.07	0.0015	113 (10.47)	82 (3.03)	87.50	**<0.0001**	57 (5.39)	17 (1.33)	31.31	**<0.0001**
Disruptive, impulse-control, and conduct disord	5 (1.37)	1 (0.49)	0.96	0.3260	18 (1.67)	24 (0.89)	4.30	0.0382	6 (0.57)	0 (0.00)	7.30	0.0069
Schizophrenia	151 (41.26)	86 (42.16)	0.04	0.8344	130 (12.05)	288 (10.64)	1.56	0.2117	83 (7.85)	59 (4.60)	10.76	0.0010
Trauma and stressor disorders	59 (16.12)	22 (10.78)	3.06	0.0803	303 (28.08)	521 (19.25)	35.37	**<0.0001**	179 (16.93)	193 (15.04)	1.55	0.2129
Substance and addictive disorders	29 (7.92)	8 (3.92)	3.46	0.0630	253 (23.45)	621 (22.94)	0.11	0.7382	89 (8.42)	70 (5.46)	8.04	0.0046
Obsessive-compulsive disorders	67 (18.31)	20 (9.80)	7.32	0.0068	76 (7.04)	176 (6.50)	0.36	0.5459	247 (23.37)	178 (13.87)	35.15	**<0.0001**
Sexual dysfunctions	8 (2.19)	0 (0.00)	4.52	0.0335	18 (1.67)	4 (0.15)	30.87	**<0.0001**	47 (4.45)	9 (0.70)	34.80	**<0.0001**
Gender dysphoria	3 (0.82)	0 (0.00)	1.68	0.1948	7 (0.65)	3 (0.11)	8.47	0.0036	2 (0.19)	0 (0.00)	2.43	0.1191
Paraphilic disorders	3 (0.82)	1 (0.49)	0.20	0.6515	2 (0.19)	3 (0.11)	0.32	0.5687	7 (0.66)	2 (0.16)	3.88	0.0489
Neurocognitive disorders	27 (7.38)	43 (21.08)	22.83	**<0.0001**	5 (0.46)	17 (0.63)	0.36	0.5475	34 (3.22)	41 (3.20)	0.00	0.9771
Bipolar disorders	35 (9.56)	27 (13.24)	1.82	0.1770	168 (15.57)	745 (27.52)	60.22	**<0.0001**	100 (9.46)	154 (12.00)	3.87	0.0491
Depressive disorders	186 (50.82)	95 (46.57)	0.95	0.3305	554 (51.34)	1653 (61.06)	29.98	**<0.0001**	686 (64.90)	889 (69.29)	5.08	0.0243
Anxiety disorders	190 (51.91)	117 (57.35)	1.56	0.2117	413 (38.28)	1586 (58.59)	127.73	**<0.0001**	787 (74.46)	1004 (78.25)	4.66	0.0310
Feeding and eating disorders	3 (0.82)	5 (2.45)	2.52	0.1125	10 (0.93)	223 (8.24)	71.40	**<0.0001**	5 (0.47)	30 (2.34)	13.69	0.0002
Elimination disorders	10 (2.73)	13 (6.37)	4.48	0.0342	6 (0.56)	39 (1.44)	5.14	0.0234	28 (2.65)	45 (3.51)	1.41	0.2346
Sleep-wake disorders	168 (45.90)	134 (65.69)	20.58	**<0.0001**	523 (48.47)	1975 (72.96)	206.11	**<0.0001**	717 (67.83)	1010 (78.72)	35.54	**<0.0001**
Dissociative disorders	0 (0.00)	4 (1.96)	7.23	0.0072	7 (0.65)	35 (1.29)	2.92	0.0876	1 (0.09)	7 (0.55)	3.46	0.0629
Somatic symptom disorders[a]	20 (5.46)	14 (6.86)	0.46	0.4992	26 (2.41)	131 (4.84)	11.46	0.0007	107 (10.12)	114 (8.89)	1.04	0.3083

Data are expressed as N (%) or the mean ± standard deviation. Bold type indicates statistical significance ($p < 0.001$), the exact value of Bonferroni correction is 0.0025 (0.05/20). Bottom line indicates that the proportion of all control groups is less than 5%, and its statistical power may be weak. [a] The proportion is not consistently predominant of one gender (male or female) across three clusters of personality disorders.

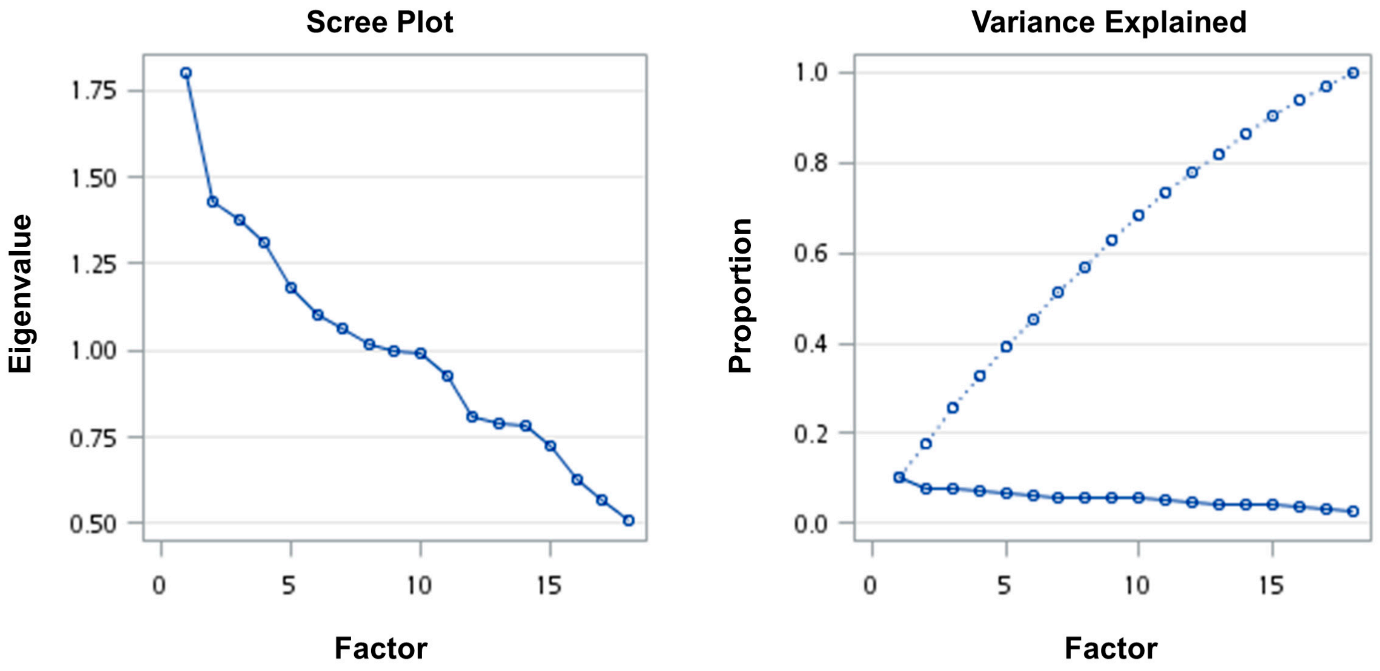

Figure S1. Scree plot and variance explained of cluster A personality disorder.

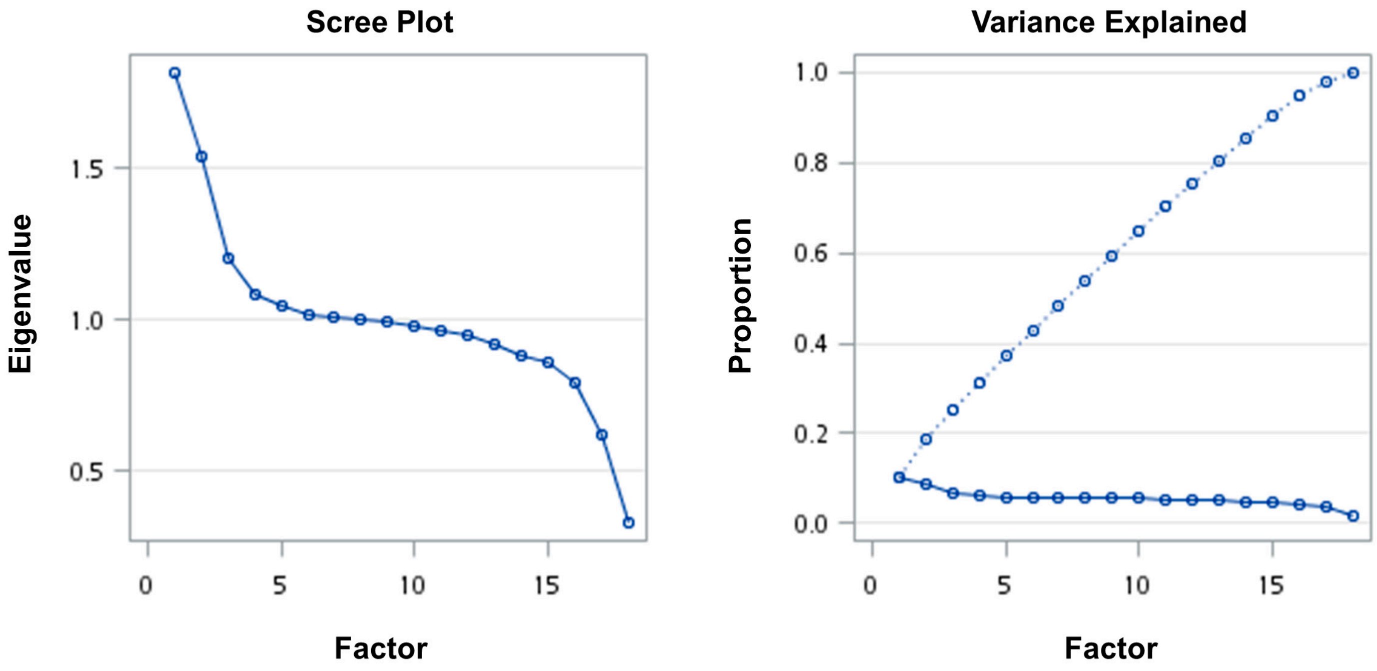

Figure S2. Scree plot and variance explained of cluster B personality disorder.

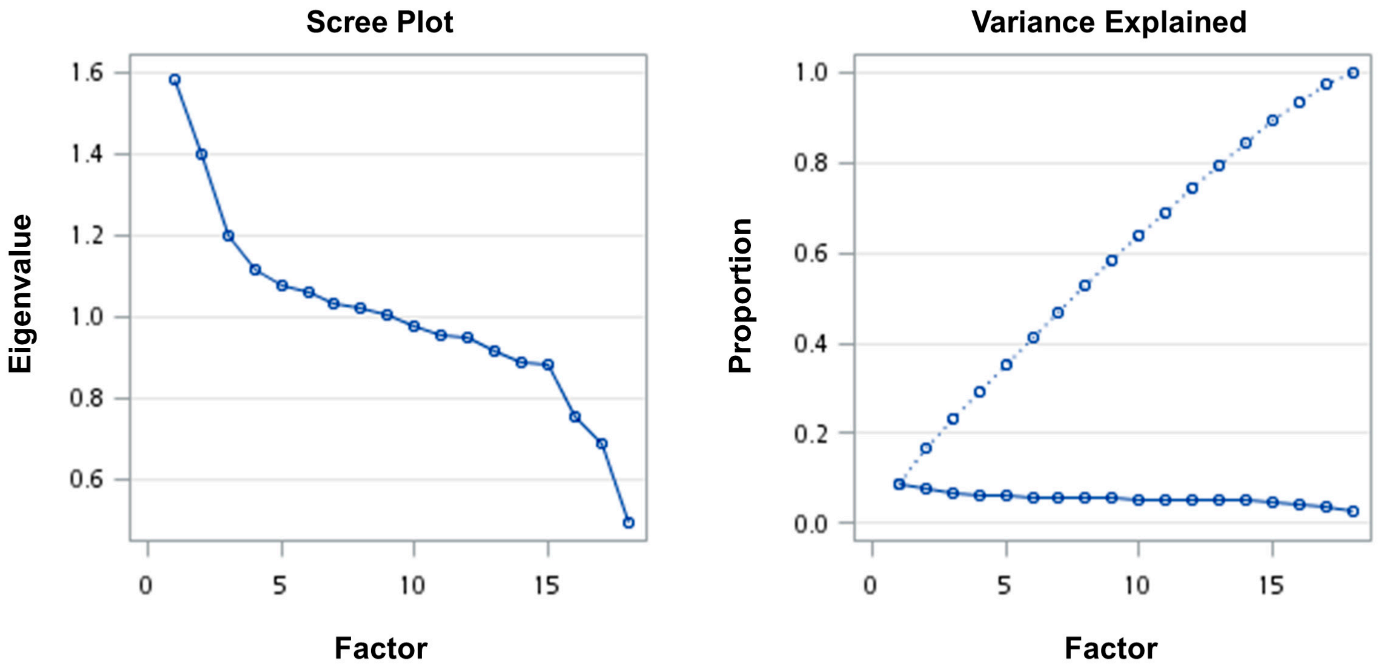

Figure S3. Scree plot and variance explained of cluster C personality disorder.

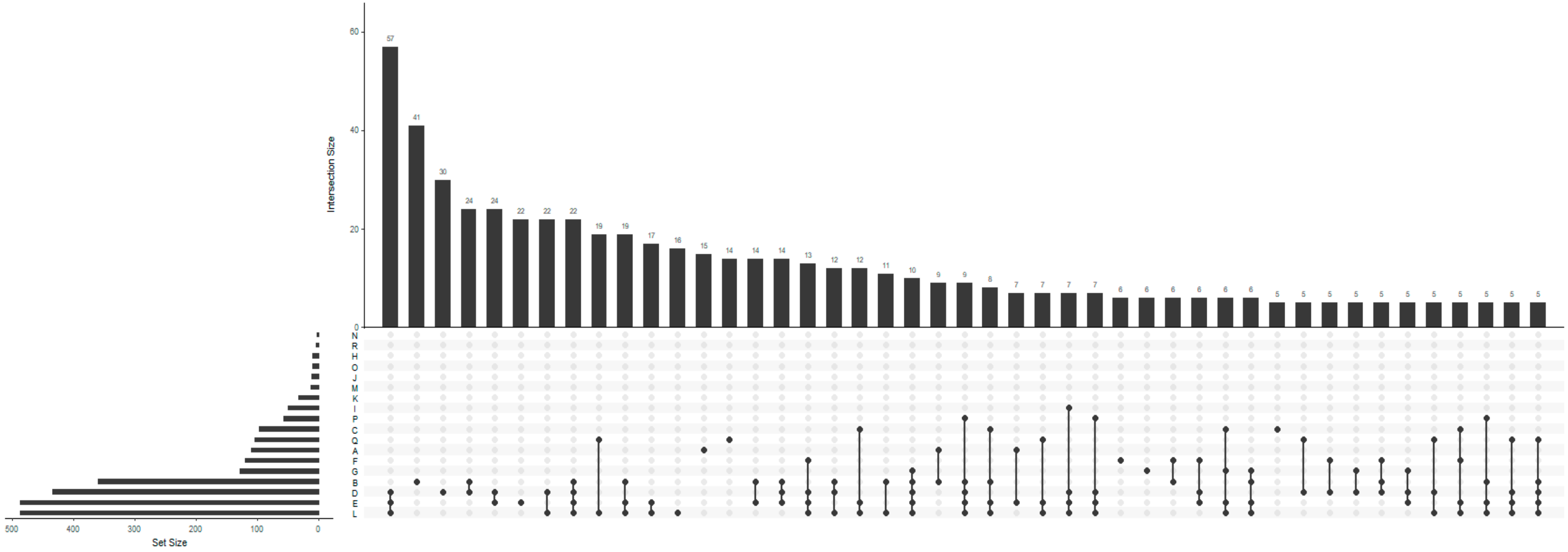

Figure S4. Upset plot of cluster A personality disorder. The Upset plot shows the part where the number of intersections is greater than 5. Abbreviation: A, Neurodevelopmental disorders; B, Schizophrenia; C, Bipolar disorders; D, Depressive disorders; E, Anxiety disorders; F, Obsessive-compulsive disorders; G, Trauma and stressor disorders; H, Dissociative disorders; I, Somatic symptom disorders; J, Feeding and eating disorders ; K, Elimination disorders; L, Sleep-wake disorders; M, Sexual dysfunctions; N, Gender dysphoria; O, Disruptive, impulse-control, and conduct disorders; P, Substance and addictive disorders; Q, Neurocognitive disorders; R, Paraphilic disorders.

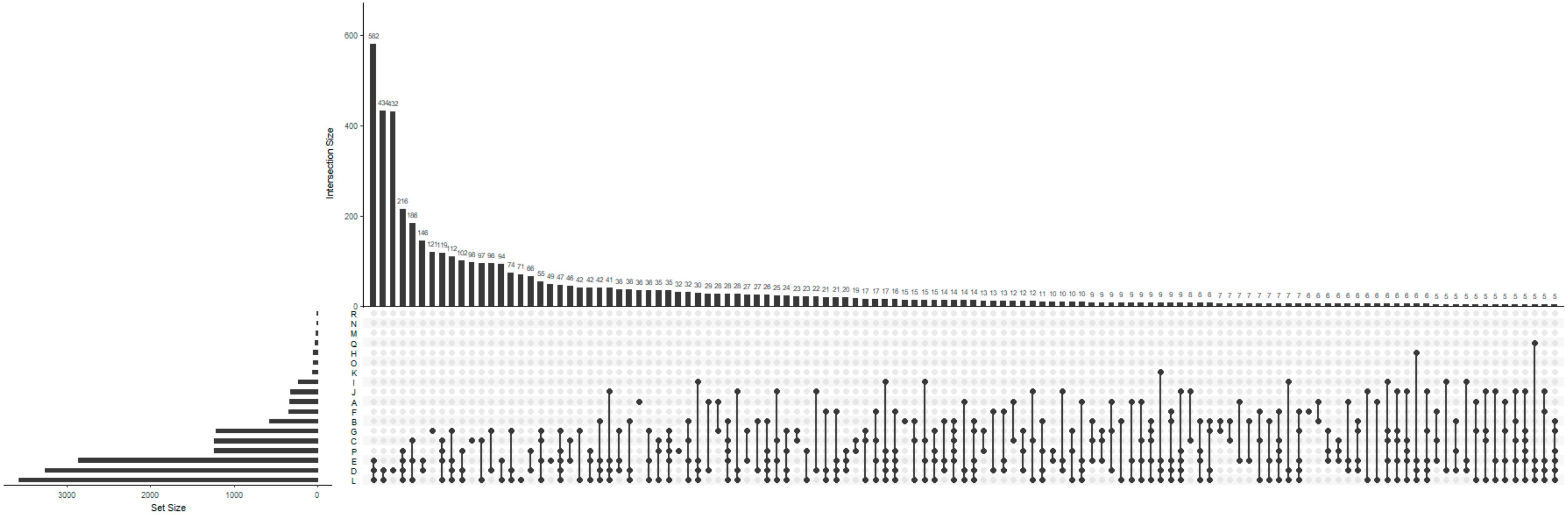

Figure S5. Upset plot of cluster B personality disorder. The Upset plot shows the part where the number of intersections is greater than 5. Abbreviation: A, Neurodevelopmental disorders; B, Schizophrenia; C, Bipolar disorders; D, Depressive disorders; E, Anxiety disorders; F, Obsessive-compulsive disorders; G, Trauma and stressor disorders; H, Dissociative disorders; I, Somatic symptom disorders; J, Feeding and eating disorders ; K, Elimination disorders; L, Sleep-wake disorders; M, Sexual dysfunctions; N, Gender dysphoria; O, Disruptive, impulse-control, and conduct disorders; P, Substance and addictive disorders; Q, Neurocognitive disorders; R, Paraphilic disorders.

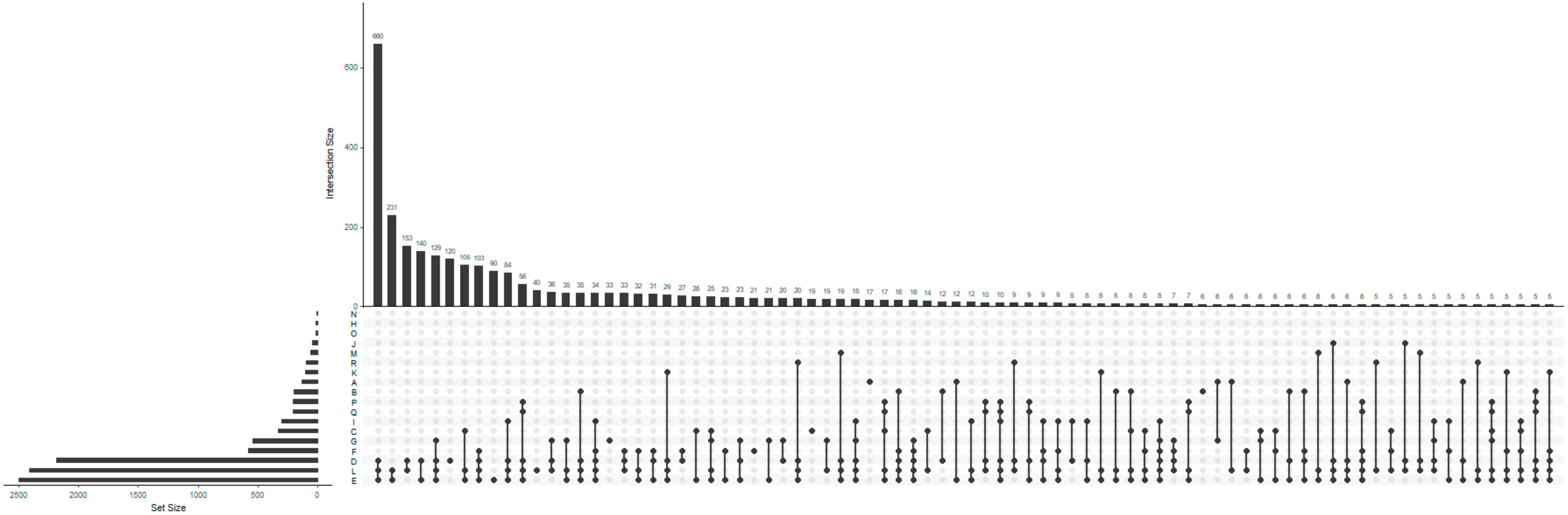

Figure S6. Upset plot of cluster C personality disorder. The Upset plot shows the part where the number of intersections is greater than 5. Abbreviation: A, Neurodevelopmental disorders; B, Schizophrenia; C, Bipolar disorders; D, Depressive disorders; E, Anxiety disorders; F, Obsessive-compulsive disorders; G, Trauma and stressor disorders; H, Dissociative disorders; I, Somatic symptom disorders; J, Feeding and eating disorders ; K, Elimination disorders; L, Sleep-wake disorders; M, Sexual dysfunctions; N, Gender dysphoria; O, Disruptive, impulse-control, and conduct disorders; P, Substance and addictive disorders; Q, Neurocognitive disorders; R, Paraphilic disorders.
